# Haplotypes of intron 4 of the estrogen receptor alpha gene and hip fractures: a replication study in Caucasians

**DOI:** 10.1186/1471-2350-11-16

**Published:** 2010-01-28

**Authors:** Javier Velasco, José L Hernández, José L Pérez-Castrillón, María T Zarrabeitia, María A Alonso, Jesús González-Macías, José A Riancho

**Affiliations:** 1 Department of Internal Medicine, Hospital U.M. Valdecilla, University of Cantabria, RETICEF. Santander, Spain; 2 Department of Internal Medicine, Hospital U. Río Hortega, University of Valladolid, RETICEF. Valladolid, Spain; 3 Unit of Legal Medicine, University of Cantabria, Santander, Spain; 4 Department of Traumatology, Hospital U.M. Valdecilla, University of Cantabria, Santander, Spain

## Abstract

**Background:**

Despite their great impact, few genetic association studies have used hip fractures as an endpoint. However, the association of two polymorphisms on intron 4 of estrogen receptor alpha (*ESR1*) with hip fractures was recently reported in a Chinese population. The aim of this study was to investigate whether such association is also present in Caucasians.

**Methods:**

We analyzed those two SNPs and another neighbour SNP located on the exon 4 of *ESR1 *in 787 patients with hip fractures and 953 controls from Spain.

**Results:**

The allelic frequencies differed markedly from those reported in Asian populations. Nevertheless, haplotypes including the rs3020314 and rs1884051 loci in intron 4 showed a significant association with hip fractures (omnibus test p = 0.006 in the whole group and 0.00005 in women). In the sex-stratified analysis, the association was significant in females, but not in males. In women, the CA haplotype appeared to have a protective influence, being present in 6.5% of the controls, but only in 3% of patients with fractures (odds ratio 0.39; 95% confidence interval 0.26-0.59; estimated population preventive fraction 3.5%). The inclusion of the rs1801132 SNP of exon 4 further increased the statistical significance of the association (odds ratio 0.17; 95% CI 0.08-0.37; p = 0.00001). Each SNP appeared to contribute independently to the association. No genotype-related differences in gene expression were found in 42 femoral bone samples.

**Conclusions:**

This study confirms the association of some polymorphisms in the region of exon 4/intron 4 of *ESR1 *and hip fractures in women. However, there are marked differences in allele frequencies between Asian and Caucasian populations.

## Background

Osteoporosis has a strong genetic component, and twin and family studies have shown that the variation of skeletal traits such as bone mineral density and bone size depends on heritable factors to a large extent [1,2]. Thus, for the past 15 years many studies have been performed in order to identify the genes responsible for such hereditary influence. Sex steroids play a critical role in bone homeostasis [3]. Hence, not surprisingly, estrogen-related genes, including enzymes involved in estrogen synthesis [4,5] and the estrogen receptor alpha gene (*ESR1*) have been widely studied. *ESR1 *is located on chromosome 6q25 and comprises 8 exons [6]. After the seminal work by Kobayashi et al [7], many investigators explored the association between *ESR1 *polymorphisms and bone phenotypes. In the majority of studies, some closely linked polymorphisms situated in intron 1, frequently characterized by using the restriction enzymes PvuII and XbaI, were analyzed. The results have been controversial, with some, but not all studies showing an association of those polymorphisms with BMD or fractures [8-12]. Nevertheless, a large meta-analyses and a multicenter study showed evidence for an association with bone mineral density (BMD) or vertebral fractures [10,11]. Recent genome-wide association (GWA) studies also showed a very significant association between certain *ESR1 *polymorphisms and BMD [13,14].

Low BMD is associated with an increased risk of fractures, but BMD by itself has limited ability to predict the overall fracture risk [15-17]. Therefore, from a clinical point of view, osteoporotic fractures, and particularly hip fractures, are a more relevant outcome than BMD. However, few genetic studies have been designed with hip fractures as an endpoint, in part due to the difficulties inherent to studies including elderly individuals. On the other hand, it has been suggested that the relative importance of genetic factors may decrease with advancing age [18,19]. However, a recently published study found a strong association of some frequent polymorphisms in the intron 4 of *ESR1 *and hip fractures in Asian population, whereas no association was found with the most widely studied polymorphisms situated in proximal regions of the gene (such as the "PvuII" locus in intron 1) [20]. The aim of this study was to replicate those results in a Caucasian population and explore the potential functional consequences by determining gene expression in the bone tissue.

## Methods

### Study subjects

This was a case-control study which included 1740 individuals over 50 years of age. Patients admitted to hospital with a hip fracture were included (n = 787, 258 men and 529 women). Those with fractures due to high-impact trauma (such as traffic accidents and falls from a height), diseases causing secondary osteoporosis (cancer, rheumatoid arthritis, malabsorption, severe systemic diseases, etc) or taking drugs known to have a deleterious effect on bone metabolism (corticosteroids, anticonvulsants) were excluded. Control subjects (n = 953, 159 men and 794 women) over 50 years of age were recruited by voice and written announcements from various sources to prevent a preferential selection bias (hospital workers, civic associations, religious groups and geriatric residences). They were selected among individuals without known osteoporotic fractures and not receiving anti-osteoporotic therapy or hormone replacement therapy, either at the time of study or in the past. All subjects were interviewed by one of the investigators in order to check the absence of exclusion criteria (as in hip fracture patients). Subjects with non-Spanish ancestry were excluded. The study protocol was approved by the Institutional Committee of Ethics in Clinical Research, and informed consent was obtained from study subjects or their representatives.

### Genotyping

DNA was isolated from the peripheral blood or from buccal swabs with a commercial kit (Qiagen, Hilden, Germany), following the manufacturer's instructions. We studied two SNPs situated in intron 4 of the *ESR1 *gene, which had been previously associated with hip fractures in the Chinese population (rs3020314 and rs1884051)[20]. We also studied the neighbor rs1801132 polymorphism, located on exon 4, which was associated with vertebral fractures in white women from the USA [21]. They were typed using Taqman assays (Applied Biosystems; Foster City, CA). Random samples were used as replicate controls to check the consistency of results. The genotyping rate was >98.5% for all three SNPs.

### Gene expression

*ESR1 *expression was determined by reverse transcription (RT)-real time quantitative PCR. Bone samples were obtained from the femoral neck during hip replacement procedures for hip fractures in 42 patients. The periosteum and the cortical bone were removed. Small trabecular fragments were extensively washed with PBS, snap-frozen in liquid nitrogen and stored at -70°C. Unthawed fragments were mashed and pulverized with a tissue homogenizer into Trizol (Invitrogen) to extract RNA. Then RNA was reverse-transcribed with the Superscript III kit (Invitrogen), using random hexamers as primers. In negative control reactions reverse transcriptase was omitted. After RT, gene expression level was determined by real-time PCR in an ABI7300 apparatus (Applied Biosystems). The reactions were performed in triplicate in 96-well plates containing aliquots of the cDNA obtained by reverse transcription, 5 μl of universal PCR master mix, and specific primers and probe complementary to exons 3-4 of *ESR1 *(Taqman Gene Expression Assays, ref. Hs00174860_m1, Applied Biosystems). The cycle threshold (Ct) was determined. This represents the cycle at which a significant increase in fluorescence is first detected and is inversely related to the amount of target cDNA in the starting material. The results were normalized to TBP (TATA box binding protein) expression analyzed in the same reaction plate, and estimated as 2^Δ^, where Δ is the difference of the TBP Ct minus the *ESR1 *Ct. We confirmed the presence of abundant transcripts of factors typical for cells of the osteoblastic lineage, such as alkaline phosphatase, osteocalcin and sclerostin in those fragments (not shown).

### Statistical analyses

The Hardy-Weinberg equilibrium, the linkage disequilibrium parameters (D' and r^2^) and the haplotypic blocks were estimated with Haploview software [22]. The association between genotypes and fractures was tested with the Cochrane-Armitage trend test and the χ^2 ^test for dominant and recessive models implemented in Plink software [23]. The association between haplotypes and fractures was tested by an omnibus χ^2 ^test, followed by testing the association of individual haplotypes, in case of a significant global test. The association was also tested after adjusting for age as a covariate by means of a likelihood ratio χ^2 ^test. Conditional haplotype analyses were used to explore the independent effect of each SNP within the haplotypic context. All these analyses were done with Plink. Permutation corrections of p-values were computed with Haploview. The non-parametric Kruskal-wallis test, implemented in SPSS software (SPSS Inc, Chicago, IL, USA) was used to analyze the differences in gene expression between subjects with different genotypes. Power calculations were done with Quanto software http://hydra.usc.edu/gxe. We estimated the study had an a priori power over 85% to find a risk ratio difference of 30% associated with polymorphisms with a minor allele frequency over 20%, under an additive genetic model.

## Results

**Figure 1:**
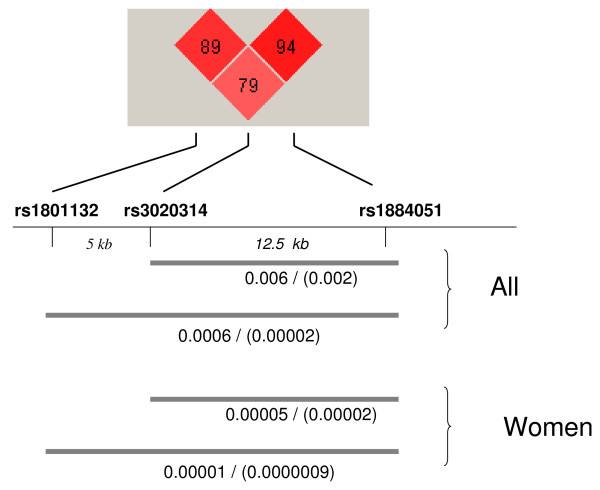
***ESR1 *haplotypes and hip fractures**. Diagram showing the association of 2-SNP (rs3020314 and rs1884051) and 3-SNP (rs1801132, rs3020314 and rs1884051) haplotypes with hip fractures in the whole group of men and women combined and in the female group. The numbers represent the p-values of the omnibus χ^2 ^test including all haplotypes and the result for the individual haplotype showing the most significant association. The upper part of the figure shows the between-locus linkage disequilibrium (D').

The mean age of patients was 83 ± 8 yr; age of controls was 74 ± 9 yr. Patients with fractures had more frequent comorbid diseases (table 1). The allelic frequencies are shown in table 2. They were similar to those reported in the Caucasian Hapmap database, but rather different from the frequencies in Asian populations. There was no evidence for departure from the Hardy-Weinberg equilibrium. The three SNPs studied were in linkage disequilibrium, with D' values between 0.79 and 0.94, and r^2 ^values between 0.44 and 0.76 (figure 1). Loci rs3020314 and rs1884051 were considered as a haplotypic block according to the 4-gamet rule.

**Table 1:** Clinical characteristics of patients and controls.

	Women		Men	
	**Controls**	**Patients**	**Controls**	**Patients**

Age	73 (9)	83 (8)	74 (10)	81 (8)
Weight^1^, kg	66 (10)	65 (12)	78 (12)	72 (8)
Height^1^, cm	154 (6)	156 (6)	166 (6)	164 (7)
Body mass index^1^, kg/m^2^	27.9 (4.2)	26.6 (4.9)	28.3 (3,6)	26.8 (5.0)
Calcium intake^2^, mg	660 (364)	631 (355)	605 (363)	579 (333)
Alcohol^3^, %	4	1	40	19
Current smokers, %	1	2	9	15
Prior stroke or TIA, %	3	9	8	19
Coronary heart disease, %	3	7	13	14
Congestive heart failure, %	5	9	3	9
Diabetes mellitus, %	13	22	15	25
Dementia, %	4	36	4	35

Numbers represent means (and SD) or percentages.

^1^Weight, height and body mass index data were available only in 41 patients with fractures

^2^Calcium intake from dairy products, mg/day

^3^Alcohol intake higher than 10 g/day

**Table 2:** Allelic frequencies in the present study and other Caucasian and Asian populations

		Present study	Hapmap-CEU	**Wang's study**[20]	Hapmap-HCB
rs1801132	C	0.82	0.82	0.52	0.49
	G	0.19	0.18	0.48	0.51
rs3020314	C	0.27	0.29	0.82	0.81
	T	0.73	0.71	0.18	0.19
rs1884051	A	0.76	0.70	0.48	0.51
	G	0.24	0.30	0.52	0.46

CEU: Utah residents with Northern and Western European ancestry; HCB: Han Chinese in Beijing. The differences between the present study and Wang's study were statistically significant for the three loci analyzed (p < 0.0001).

In the whole group there was a non-statistically significant trend for association between hip fractures and the rs1801132 (p = 0.09 and 0.05 for the codominant and recessive models) and rs3020314 (p = 0.09 and 0.06 for the codominant and recessive models) polymorphisms. In women, the three polymorphisms showed a marginally significant association with fractures in the unadjusted analysis (rs1801132, p = 0.03; rs3020314, p = 0.04; rs1884051, p = 0.03), but it was no longer significant (p > 0.2) after including age as a covariate. The results were not statistically significant in the male group.

However, the haplotypic analysis revealed a strong association between gene variations and fracture risk, in the whole group and in the female group (figure 1). Haplotypes including rs3020314 and rs1884051 loci showed a significant association (omnibus test p = 0.006 in the whole group and 0.00005 in women). The inclusion of the rs1801132 locus further increased the statistical significance of the association (figure 1). The results remained significant after taking into consideration the error inflation by permutation analyses. Permutation p-values were 0.008 and 0.0002, for the 2-SNP (rs3020314- rs1884051) and the 3-SNP analyses, respectively. In the conditional analysis, all three SNPs appeared to contribute significantly to the association of the haplotypes with fractures (all p-values < 0.007). The association of individual haplotypes with fractures is shown in table 3. It was driven by the CA haplotype, as when this haplotype was controlled for the remaining haplotype set was no longer associated with fractures in the omnibus test. The CA haplotype appeared to have a protective role, with a frequency of 6.5% in the control group and 3% in the fracture group. Similarly, the frequency of the 3-SNP GCA haplotype was 3.2% in the control group and 0.7% in the fracture group.

**Table 3:** Association of *ESR1 *haplotypes with hip fractures in women.

	Frequency in cases (%)	Frequency in controls (%)	OR (95% CI)	p-value
**2-SNP**				
CG	23.6	20.1	1	-
TG	0.8	1.2	0.54 (0.26-1.16)	0.22
CA	3.0	6.5	0.39 (0.26-0.59)	1.8 × 10^-5^
TA	72.6	72.2	0.85 (0.70-1.0)	0.77

**3-SNP**				
GCG	14.7	13.5	1	-
CCG	8.9	6.8	1.18 (0.83-1.69)	0.05
CTG	0.8	1.2	0.58 (0.27-1.25)	0.22
GCA	0.6	3.2	0.17 (0.08-0.37)	8.6 × 10^-7^
CCA	2.4	3.3	0.67 (0.4-1.13)	0.13
GTA	1.4	1.9	0.65 (0.32-1.31)	0.26
CTA	71.2	70.1	0.92 (0.73-1.17)	0.52

2-SNP haplotypes include rs3020314 and rs1884051

3-SNP haplotypes include rs1801132, rs3020314 and rs1884051

Since the average age was somewhat higher in the fracture group than in the control group, we repeated the haplotypic analyses including age as a covariate. The association between estrogen receptor haplotypes and fractures remained statistically significant after controlling for age (p = 0.03 and p = 0.02 for the 2-SNP and the 3-SNP haplotype omnibus tests, respectively). We also performed an association analysis including only individuals of 70-85 years of age in order to get age-matched patient and control groups. This subgroup included 520 fractured patients (age 79 ± 4) and 376 controls of similar age (age 78 ± 5). As in the previous analysis, there was a significant association between the 2-SNP and 3-SNP haplotypes and fractures in men and women combined and in the female group. The 3-SNP omnibus test showed p-values of 0.02 and 0.001, for the whole subgroup and for women, respectively. The p-values for the most significant haplotype (GCA) were 0.001 and 0.0001, respectively. In men, the omnibus test p-value was 0.17. A Bayesian analysis did not reveal any suggestion of a plausible association between haplotypes and fractures in the male group (not shown). Despite their similar age, patients of 70-85 years of age had a higher frequency of comorbid diseases than the control group, particularly stroke, diabetes mellitus and dementia. Nevertheless, when women with those frequent diseases were excluded, the estrogen receptor haplotypes remained significantly associated with a reduced risk of fractures (CA haplotype OR 0.41, p = 0.011; GCA haplotype OR 0.19, p = 0.027).

**Figure 2:**
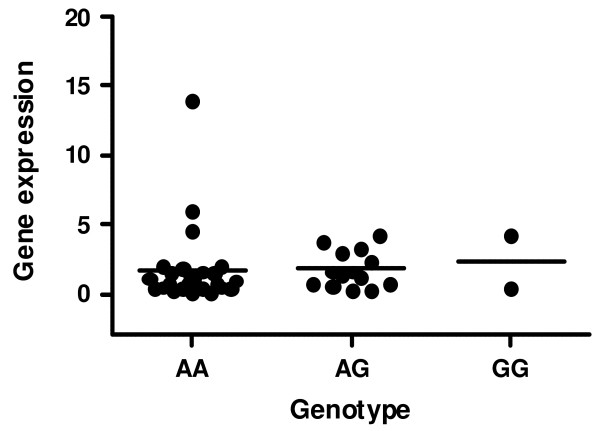
***ESR1 *alleles and expression**. Abundance of estrogen receptor gene transcripts in bone samples from patients with hip fractures according to the rs1884051 genotypes (n = 26, 14 and 2). The results are expressed as arbitrary units, normalized by the housekeeping gene TBP.

Gene expression was studied in 42 bone samples. We found no significant differences in the abundance of estrogen receptor transcripts across different genotypes. The results for the rs1884051 genotypes are shown in figure 2. Similar results were found when gene expression was analyzed in relation to rs3020314 and rs1884051SNP alleles (not shown). The sample size did not allow to analyze the relationship between haplotypes and gene expression.

## Discussion

Wang et al recently performed an *ESR1 *gene-wide association study in a moderate-size group of Chinese men and women, including 350 patients with hip fractures and 350 controls [20]. They found that a common haplotype in intron 4, present in 57% of control subjects, was associated with a decreased risk of hip fractures, with and odds ratio of 0.68 (confidence interval 0.55-0.84). Hip fractures are among the most devastating osteoporotic fractures. Therefore, the findings in Wang's report were of great scientific (and possibly practical) interest.

Our study, aimed to replicate those findings in Caucasians, confirmed a strong association of the haplotypes of the intron 4 of *ESR1 *with hip fractures, particularly in women. However, the allelic frequencies of the SNPs involved, and consequently the haplotype frequencies, were rather different from those found in Chinese. Allelic frequencies were less balanced in our population, and the frequency of the haplotypes influencing fracture risk was much lower (6.5%, versus 57% in the Chinese). Therefore, their impact at the population level must also be lower in Caucasians than in the Asians. Although we cannot estimate the true incidence in a case-control study like this, assuming a lifetime frequency of hip fractures about 15%, it could be estimated that the population preventive fraction due to the estrogen receptor haplotype would be about 3.5%. The differences in the allelic frequency distributions are not unique to our studies. According to Hapmap data, they reflect the differences between the general Caucasian and Asian populations. We also genotyped a nearby SNP located on exon 4, about 5 kb upstream from those reported by Wang, which has been associated with vertebral fractures in a candidate gene study of white American women from the SOF cohort. The inclusion of this SNP in the haplotypic analysis resulted in a stronger association signal.

The incidence of hip fractures increases exponentially with age. Given the difficulty to recruit suitable very old controls, our control and fracture groups were not well age-matched. On the other hand, the frequency of comorbid disorders was higher in the fracture group, independently of age. These represent potential limitations of the study. In general, the younger age of the controls would be expected to bias our study towards the null result (as some of the control individuals may suffer a fracture in later years). However, other type of bias could theoretically exist if, for instance, the polymorphisms were associated with life expectancy. Therefore, after the crude analysis we performed both an age-adjusted analysis and an analysis of the association including only patients and controls of similar "middle" age. Both analyses confirmed the association between *ESR1 *haplotypes and hip fractures. Bone mineral density is a well-known risk factor for fractures, but other factors, including body weight, are also important. We could not establish to what extent the *ESR1 *haplotypes association with fractures was dependent on those factors because we have BMD and anthropometric data in only a minority of patients with fractures. As in other studies [24], we found a higher frequency of comorbid diseases in the fracture group than in the control group, particularly cerebrovascular diseases, dementia and diabetes mellitus. However, the association of *ESR1 *haplotypes with fractures appeared to be independent of those comorbidities.

The mechanisms explaining this association are unclear. The conditional analyses suggested that each of the three SNPs had an independent significant contribution to the association. Therefore, our study does not allow to identify a causal SNP. Since rs11801132 is a synonymous polymorphism and the two other polymorphisms are located in an intronic region, changes in the aminoacid sequence do not explain the association with hip fractures. Allele-related differences in gene expression are a more likely explanation. However, we have not been able to show genotype-related differences in estrogen receptor transcripts in bone tissue. Nevertheless, given the number of bone samples, the study did not have enough power to analyze possible differences in gene expression at the haplotypic level. In the Chinese population, Wang found that the CC haplotype (rs3020314-rs1884051) was associated with a decreased fracture risk (OR 0.68; 95% confidence interval 0.55-0.84). They designed the rs1884051 polymorphism according to the alleles in the reverse DNA strand. Therefore, that haplotype corresponds to the CG haplotype in our study. Interestingly, we did not observe an effect of such haplotype on fracture risk, but we did find a protective effect of the CA haplotype (odds ratio 0.49; 95% confidence interval 0.26-0.59). Taking together the different haplotypes involved, the differences in allelic and haplotype frequencies in the Chinese and Caucasian populations and the lack of differences in gene expression at the allele level suggest that the association with fracture risk is not directly due to the SNPs included in these studies, but to other polymorphisms in linkage disequilibrium.

## Conclusions

In conclusion, we have confirmed a significant association of genetic variants in the region of exon 4-intron 4 of the *ESR1 *and hip fractures in the Caucasian women. This and previous results in Asian population suggest that, regarding hip fractures, the variation along this region may be more important than the most widely studied proximal region of the gene. Given the devastating consequences of these fractures, further studies are warranted to identify the causal polymorphisms and to elucidate the molecular mechanisms responsible for such association. On the other hand, it may be interesting to explore if the differences in the allelic frequency distributions between Asian and Caucasian populations are related to the worldwide regional differences in the incidence of hip fractures.

## Competing interests

The authors declare that they have no competing interests.

## Authors' contributions

JV, JLH, JLPC and MAA recruited and examined cases and controls and collected the clinical data. MTZ was responsible for the genotyping. JGM contributed to the study design and data interpretation. JAR designed the study, analyzed the data and wrote the first manuscript draft. All authors read and approved the final manuscript.

## Pre-publication history

The pre-publication history for this paper can be accessed here:

http://www.biomedcentral.com/1471-2350/11/16/prepub
